# Cellular migration, transition and interaction during regeneration of the sponge *Hymeniacidon heliophila*

**DOI:** 10.1371/journal.pone.0178350

**Published:** 2017-05-25

**Authors:** Cristiano C. Coutinho, Ivone de Andrade Rosa, John Douglas de Oliveira Teixeira, Leonardo R. Andrade, Manoel Luis Costa, Claudia Mermelstein

**Affiliations:** Institute of Biomedical Sciences, Federal University of Rio de Janeiro – UFRJ, Rio de Janeiro, Rio de Janeiro, Brazil; University of Colorado Boulder, UNITED STATES

## Abstract

Sponges have a high capacity for regeneration and this process improves biomass production in some species, thus contributing to a solution for the biomass supply problem for biotechnological applications. The aim of this work is to characterize the dynamics of cell behavior during the initial stages of sponge regeneration, using bright-field microscopy, confocal microscopy and SEM. We focused on the first 20 h of regeneration, during which blastema formation and epithelium initialization occur. An innovative sponge organotypic culture of the regenerating internal region is described and investigated by confocal microscopy, cell transplantation and vital staining. Cell-cell interaction and cell density are shown to affect events in morphogenesis such as epithelial/mesenchymal and mesenchymal/epithelial transitions as well as distinct cell movements required for regeneration. Extracellular matrix was organized according to the morphogenetic process observed, with evidence for cell-signaling instructions and remodeling. These data and the method of organotypic culture described here provide support for the development of viable sponge biomass production.

## Introduction

Sponges are sessile filter-feeding invertebrates that originated around 600 million years ago, according to fossil records, cellular and genetic evidence [1–4]. Sponges originated in a microorganism world and have been co-evolving with them since that time. The chemical interactions and cell-signaling processes between sponge cells and viruses, archaea, bacteria, protozoa and fungi have become very complex, leading to a rich and diverse secondary metabolism in sponges, which contain several bioactive compounds and biomaterials of biotechnological interest [5–8]. Nevertheless, to use sponges for commercial use, high amounts of sponge biomass are required [9–12]. One possibility of producing sponge biomass is to grown sponge explants *in vitro*. These explants are small fragments (1–5 cm^3^) taken from adult sponges and used to initiate sponge farming [10,13]. It has been shown that the healing and regeneration process that occurs after sponge fragmentation induces biomass production with growth rates many times greater than the undisturbed growth rate of the intact organism [14]. There is still a lack of information on the cellular and molecular events driving the regeneration process in sponges.

A complete manual for sponge morphology and definition of terms has been published by Boury-Esnault and Rützler [15]. The cellular components and morphology of the sponge *Hymeniacidon heliophila* are briefly described below to facilitate understanding of tissue regeneration. The pinacocyte is a flattened cell type with epithelial morphology. It covers the external (exopinacocyte) and internal (endopinacocyte) surfaces of the sponge body, thus forming respectively the exopinacoderm of the ectosome (cortex) and the internal endopinacoderm surface such as that which lines water channels. The ostia are openings in the exopinacoderm through which seawater enters the sponge, allowing for nutrition, respiration and reproduction of sponge cells. The basopinacoderm is an epithelial cell layer contacting the substratum to affix the sponge to a solid support, by secretion of a collagenous matrix. In *Hymeniacidon heliophila*, water channels are abundant, randomly organized and perfuse the entire sponge, with several choanocyte chambers distributed along the channels (choanoderm) [16]. These choanocyte chambers are spherical structures formed from epithelial flagellated choanocytes, cells that are involved in many processes, including feeding, reproduction and formation of stem cells. Cells with mesenchymal morphology are part of the mesohyl, the region enclosed by epithelial structures (pinacoderm and choanoderm). The archaeocytes have mesenchymal spherical, amoeboid or fusiform morphology, and together with choanocytes, they are part of the sponge stem cell system, with the totipotent ability to generate all sponge cell types (reviewed in [17–19]). Other cells with mesenchymal phenotype are not easily distinguished, but can be confirmed by their specific functions, such as sclerocytes that secrete silica for spicule extension (demosponge), and lophocytes/cholencytes/spongocytes that synthesize sponge collagen fibers.

Sponge regeneration involves the re-establishment of the natural tissue organization, with differentiated cell types acting in coordination. Sponges have an extraordinary ability for regeneration, and indeed this is part of their strategy for propagation. Dissociated sponge cells can undergo species-specific aggregation, which gives rise to new individuals when conditions are favorable. Alternatively, viable resistant bodies may form, known as primmorphs. They are characterized by external layer covered by a continuous pinacoderm, and a central zone composed primarily of spherulous cells, with the ability to generate a new organism [9,20–24]. Different conditions have been tested by different researchers and there is no standard methodology to propagate sponges by fragmentation (reviewed in 11), nor is there a uniform pattern or rate of regeneration [25–28].

Despite the diversity of regeneration processes, remodeling is a common process among different species of the Demospongiae class. It is defined by Simpson [29] as a spontaneous process of disorganization and reorganization, mainly with the disappearance of the choanosome, the region with a high frequency of choanocyte chambers and water channels. This remodeling can be compared with blastema formation, as proposed by Needham [30]. Borisenko and colleagues [17] also used the term blastema for the initial step of sponge regeneration. It defines the transitory undifferentiated mesenchyme-like cell mass that accumulates at the regenerating region and can form the missing structures by subsequent cell differentiation. They suggested the following common steps for regeneration in encrusting Demosponges: (I) closure of tissue injury and disintegration of the structures in adjacent areas; (II) formation of an undifferentiated cell mass (blastema); (III) epithelialization of the wound surface and (IV) reorganization of the inner damaged structures.

We focused our investigation at the stage of blastema formation and initial epithelialization in sponges using an innovative organotypic *in vitro* culture of the internal region of *Hymeniacidon heliophila*. With this culture, we characterized the morphogenetic process and analyzed specific cell types and sponge structures, using specific staining for nuclei and extracellular matrix components, time-lapse video microscopy by confocal microscopy and scanning electron microscopy (SEM). We found that cell-cell interactions modulated tissue morphogenesis during sponge regeneration. Our results indicated cellular transitions such as epithelial to mesenchymal and mesenchymal to epithelial phenotypes. We also found different cellular movements associated with different cell densities. Our method of organotypic culture is thus suggested as a model to further investigate sponge cell processes in future work. This method could contribute to the development of new strategies for large-scale sponge production.

## Results

### Histomorphology of natural living *Hymeniacidon heliophila*

**Fig 1:**
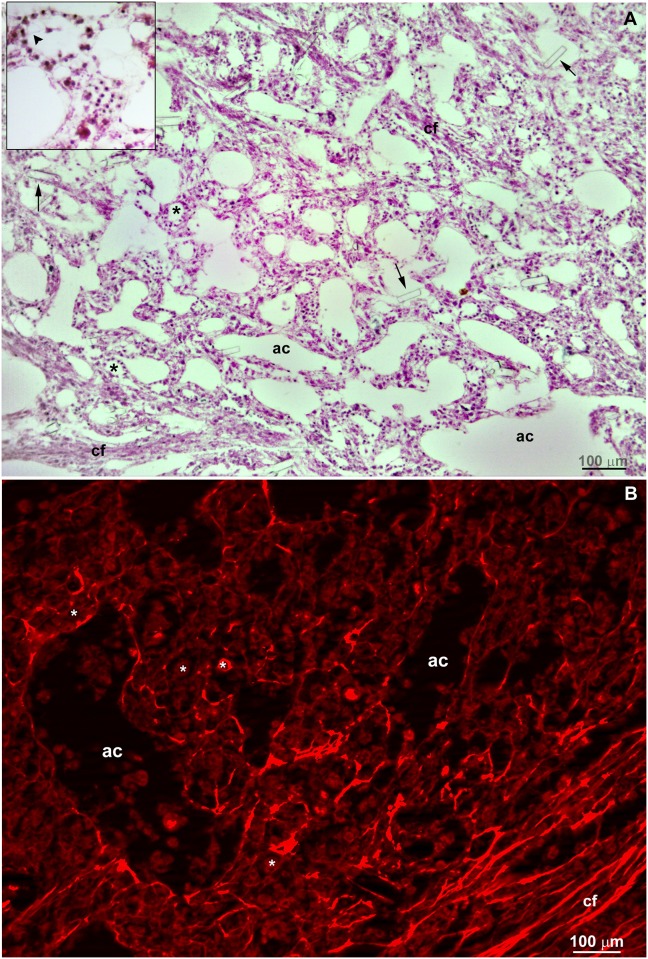
Histological sections from a natural living *Hymeniacidon heliophila*. **A)** Representative structures are indicated in 7 μm-sections stained with hematoxylin-eosin showing the choanosome with random organization of choanocyte chambers (*), aquifer channels (ac) and sparse collagen fascicles with associated fusiform aligned cells (cf). The inset shows a choanocyte chamber sectioned at the center with an apparent choanocyte flagellum (arrowhead) toward the center of the chamber. Arrows point to fragments of spicules that were generated after the sectioning of the sponge samples. **B)** Picrosirius staining revealing the ubiquitous presence of collagen with sparse collagen fascicles (cf) in the endosomal region. A bright red staining was also observed bordering the aquifer channels (ac) and choanocyte chambers (*).

Hematoxylin/eosin and picrosirius staining were used to delineate the overall morphology of cells and collagen fibers, respectively, from *Hymeniacidon heliophila* collected in the natural environment. As expected, epithelial cells (pinacocytes) were observed lining aquifer channels (endopinacoderm) and forming choanocyte chambers, whereas cells with mesenchymal phenotype filled the mesohyl (Fig 1a). Water channels with varied calibers (from 40 to 700 μm,) and choanocyte chambers were randomly distributed (Fig 1a). The mesohyl had sparse fascicles associated with aligned fusiform cells (Fig 1a). Picrosirius staining confirmed the presence of aligned collagen fibers forming these fascicles (Fig 1b). Some spicule fragments were generated after sectioning for histological preparation (Fig 1a).

### Morphological characterization of *Hymeniacidon heliophila* organotypic culture

**Fig 2:**
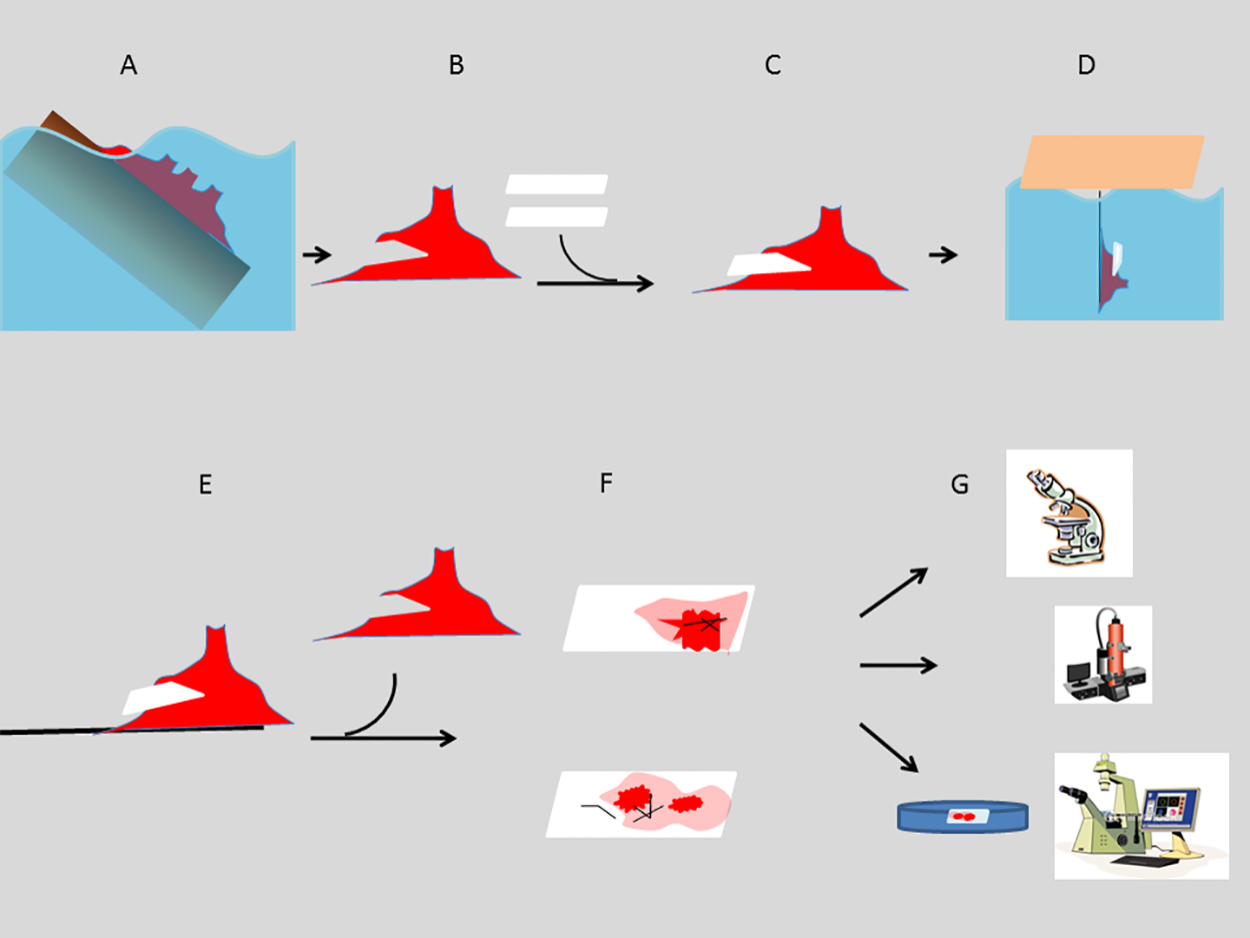
Schematic representation of the methodological steps for the sponge regeneration model. **A)** Naturally occurring *Hymeniacidon heliophila* at the intertidal zone of the collecting point. **B)** Explant is collected and a sharp blade is used for a transverse cut in relation to the erect chimneys, fistules and uneven digitations. **C)** Two apposed coverslips are introduced into the cut. **D)** This sandwich of sponge and coverslips was suspended for at least four days on a piece of Styrofoam floating in the bay close to the original collecting point. **E, F)** At the lab, the explant is opened and the two coverslips are removed from inside with internal sponge tissue attached over the area in previous contact. This was considered time 0 for regeneration, representing the initial stage and the normal histomorphology. The two areas over the coverslips with different color intensity represent basopinacoderm epithelial cell layer (light color) and dense regions with mesenchymal/amoeboid/spherical cells (dark color) and spicules (black lines). **G)** One coverslip is immersed in a Petri dish with fresh seawater (50 ml), mounted with a thin glass bottom for observation by inverted optical microscopy. Time-lapse video microscopy was recorded for the maximum time of 16.5 hours, with 1 photo every 10 min. Alternatively, regenerating tissue over the coverslips was investigated by SEM and histochemistry.

**Fig 3:**
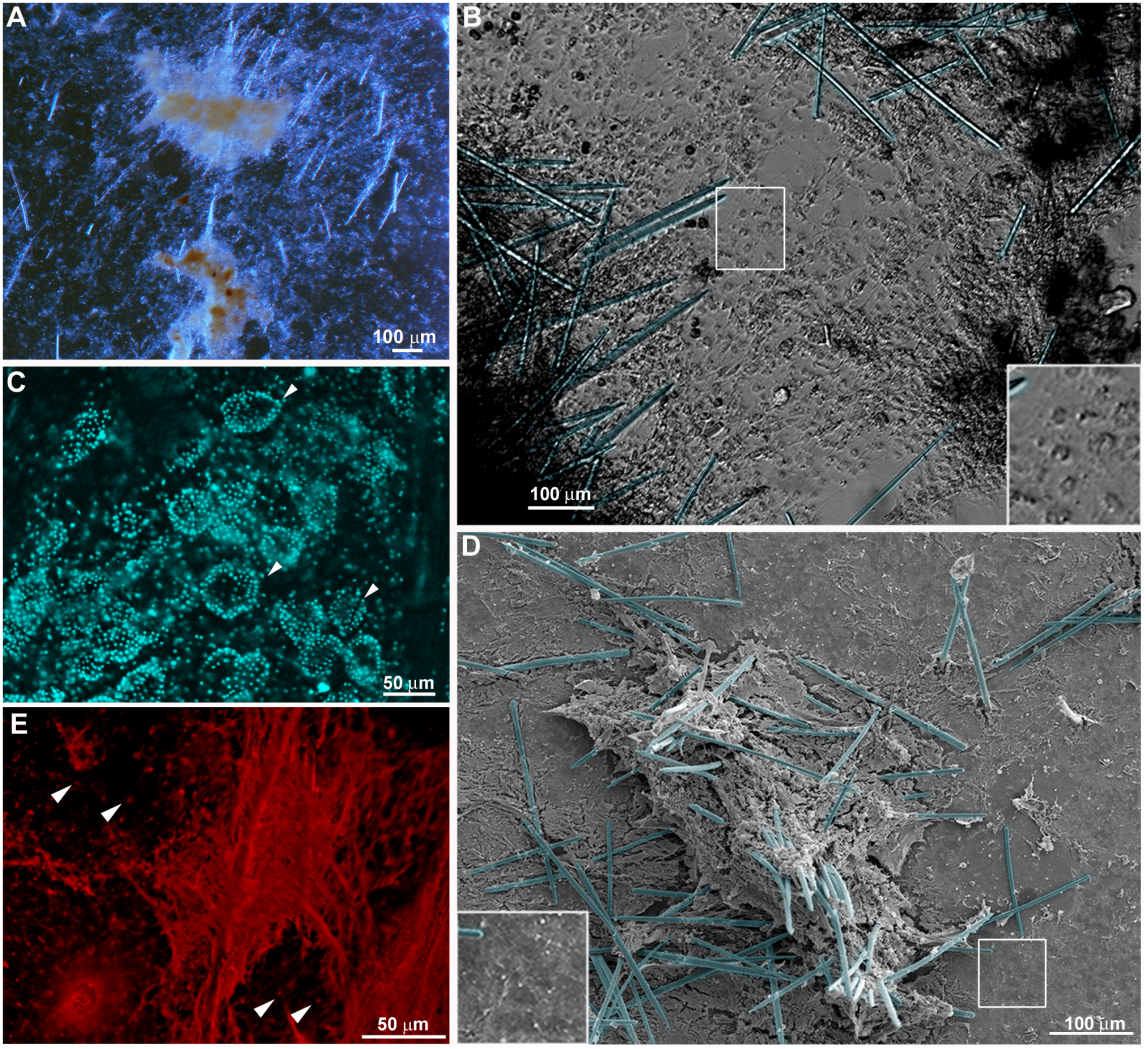
Organotypic culture at the initial stage (T0). **A)** Endosomal tissue observed by low magnification showing mineral skeleton, spicules, and distinct regions with varied tissue thickness on the coverslips. **B)** DIC of a representative field showing the basopinacoderm (gray at the center) and two flanking regions with high density of cells with mesenchymal phenotype (dark) and mineral spicules (blue). **C)** A representative field of the organotypic culture stained by DAPI to show the choanocyte chambers (arrowheads) in a region of high cell density. **D)** Representative field of the organotypic culture observed by SEM, showing a high density of mesenchymal cells and mineral spicules (blue) at the center and the basopinacoderm with some cells with mesenchymal phenotype and spicules at the border. **E)** Collagen fibers stained by Picrosirius in a high-density region of an organotypic culture. The adhesion spot of each basopinacocyte is present in the background (indicated by arrowheads).

The sponge organotypic culture was initiated when a glass coverslip was removed from the explant (T0) and fragments of endosomal tissue remained attached to it (see Fig 2 for a schematic view of the methodology used for the sponge regeneration model). The overall morphology of the initial sponge tissue culture (T0) was characterized by bright-field microscopy and SEM. The epithelial basopinacoderm cells were attached to the coverslips, while cells with mesenchymal phenotype overlay the basopinacoderm (Fig 3a, 3b and 3d). Regions with thick sponge tissue displayed choanocyte chambers, as observed by DAPI staining (DNA specific probe, Fig 3c). Silica spicules were observed all over the sponge tissue (Fig 3a, 3b, 3d and 3e). While most regions showed an unorganized distribution of spicules, in other regions where spicules were sparse, they were organized in parallel bundles (Fig 3a). Picrosirius staining revealed fascicles of parallel oriented collagen fibers associated with aligned spicules. Widespread diffuse collagen organization in mesohyl was also noticed (Fig 3e). Presence of collagen in cement for the adhesion of basopinacocytes was also evident after Picrosirius staining (Fig 3e).

**Fig 4:**
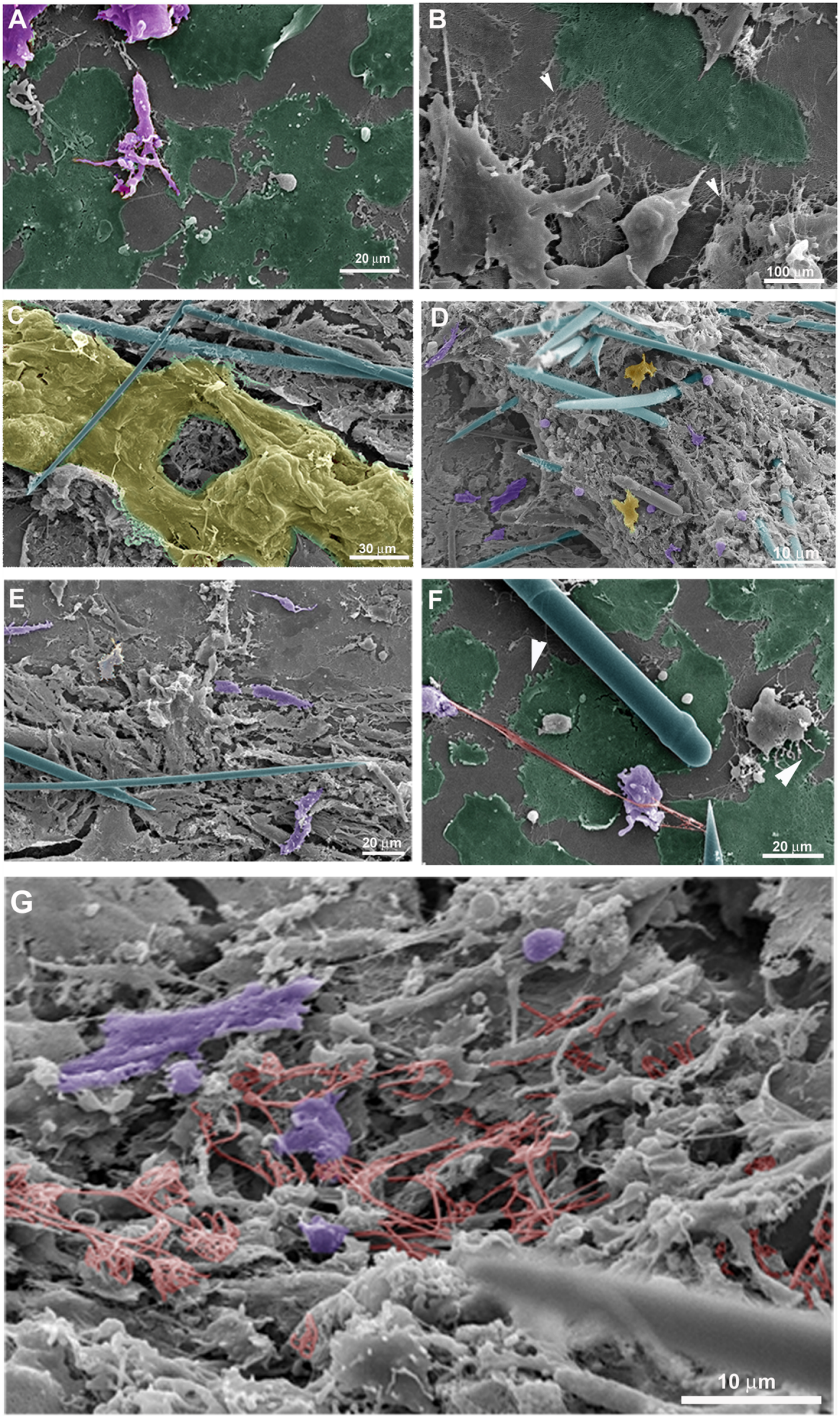
Fine structure of an organotypic culture at the initial stage (T0), visualized by SEM. **A)** Basopinacocytes (green) and mesenchymal cells (purple) with filopodia. **B)** Detailed structure of a basopinacocyte (green) showing anchoring fibers at the basopinacoderm (arrowheads). **C)** Endopinacocyte forming endopinacoderm (yellow). Spicules are shown in blue. **D)** Endosomal tissue with spicules (blue), round (light purple) and fusiform (dark purple) cells with mesenchymal phenotype with filopodium and isolated pinacocyte (yellow). **E)** Endosomal region dominated by fusiform mesenchymal cells (purple). Spicules are in blue. **F)** Basopinacocytes with filopodia (arrowheads) and some cells with mesenchymal phenotype over them. A long, thick collagen fiber (red) links two cells with mesenchymal phenotype (purple) and a spicule (blue). Basopinacocytes are represented in green. **G)** Mesh of thin collagen fibers (red) surrounding round and fusiform mesenchyme-like cells (purple).

The fine features of incipient sponge organotypic cultures at time 0 of regeneration were studied by SEM. As expected, the main cell types observed were classified as cells with epithelial phenotype or cells with mesenchymal phenotype. Flattened epithelial cells (ranging from 20 to 40 μm in diameter) resembling the basopinacoderm presented numerous membrane projections resembling filopodia (Fig 4a). Some of the membrane projections resembled cytonemes, which are membrane projections with signaling function [31]. Nanometric fibers forming a mesh were observed at the periphery of the cells anchoring the epithelium (Fig 4a and 4b). Other flattened cells formed an endopinacoderm (Fig 4c) or were isolated into the mesohyl (Fig 4d). Cells with mesenchymal phenotype overlaid the basopinacoderm and showed morphological heterogeneity, from spherical to fusiform shape and varying in length from 3 to 30 μm, suggesting the presence of different cell types (Fig 3a–3g). Most cells with mesenchymal phenotype displayed several filopodia and lamellipodia (Fig 4a–4g). Round cells showed a more homogeneous surface. Extracellular matrix elements among cells with mesenchymal phenotype were clearly visible under SEM. Two different patterns of extracellular matrix distribution were evident: 1) long, isolated thick fibers, ranging from 250 to 500 nm in diameter (Fig 3f); and 2) thinner fibers of 100 nm forming a 3-dimensional (3D) reticular meshwork containing interlaced collagen fibrils organized into firm tracts (described later) (Fig 4g). All mesenchymal cells were trapped in this 3D network of firm extracellular matrix tracts.

### Regeneration

**Fig 5:**
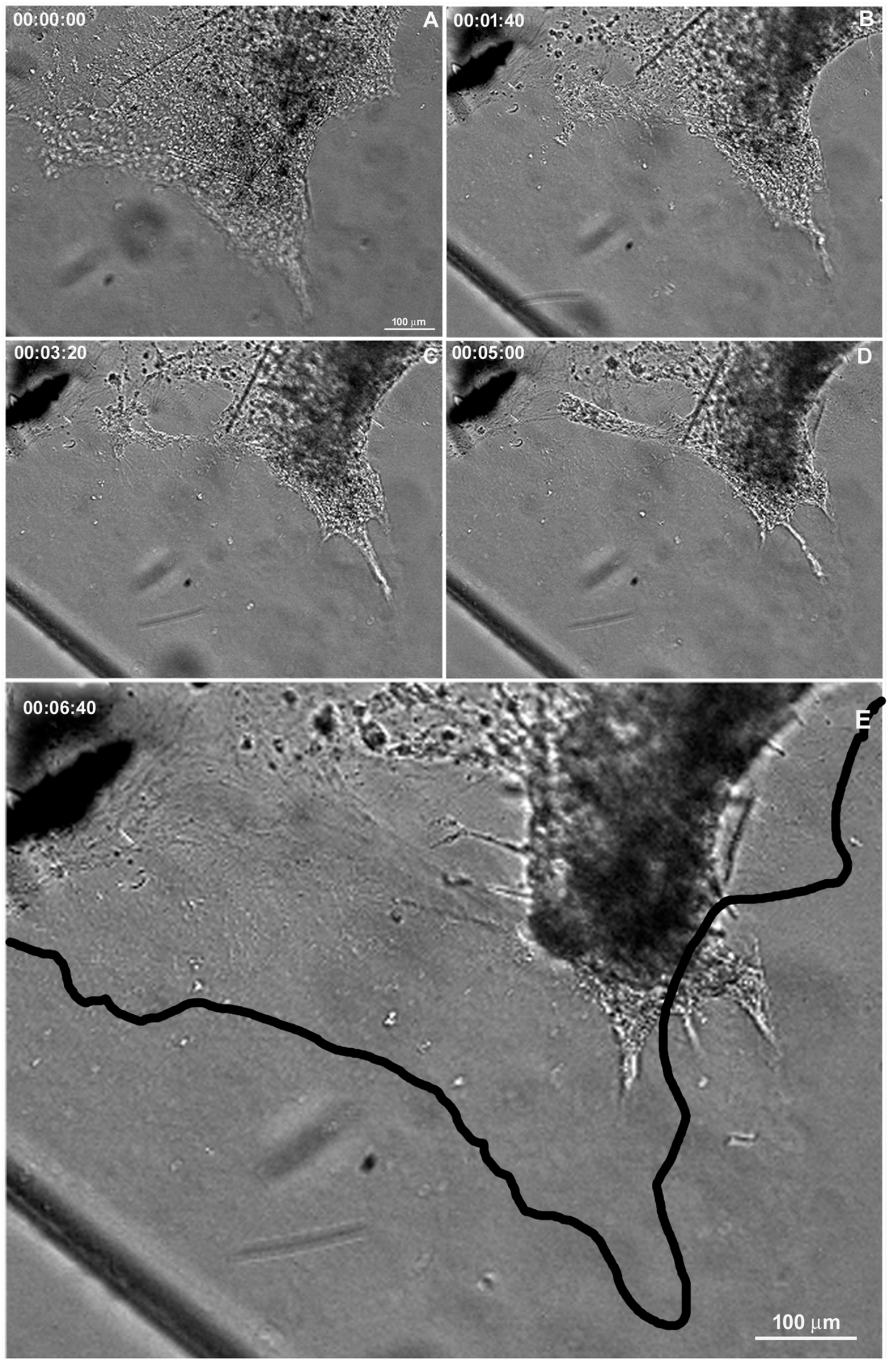
Cell movement during regeneration. Serial images from time-lapse video microscopy of the initial regeneration stage showing contraction of the organotypic culture. The area of the sponge tissue at the initial stage was outlined and projected onto the image at the final stage (black line in E) to show the tissue contraction. The regeneration time is shown in each stage (see S1 Fig).

**Fig 6:**
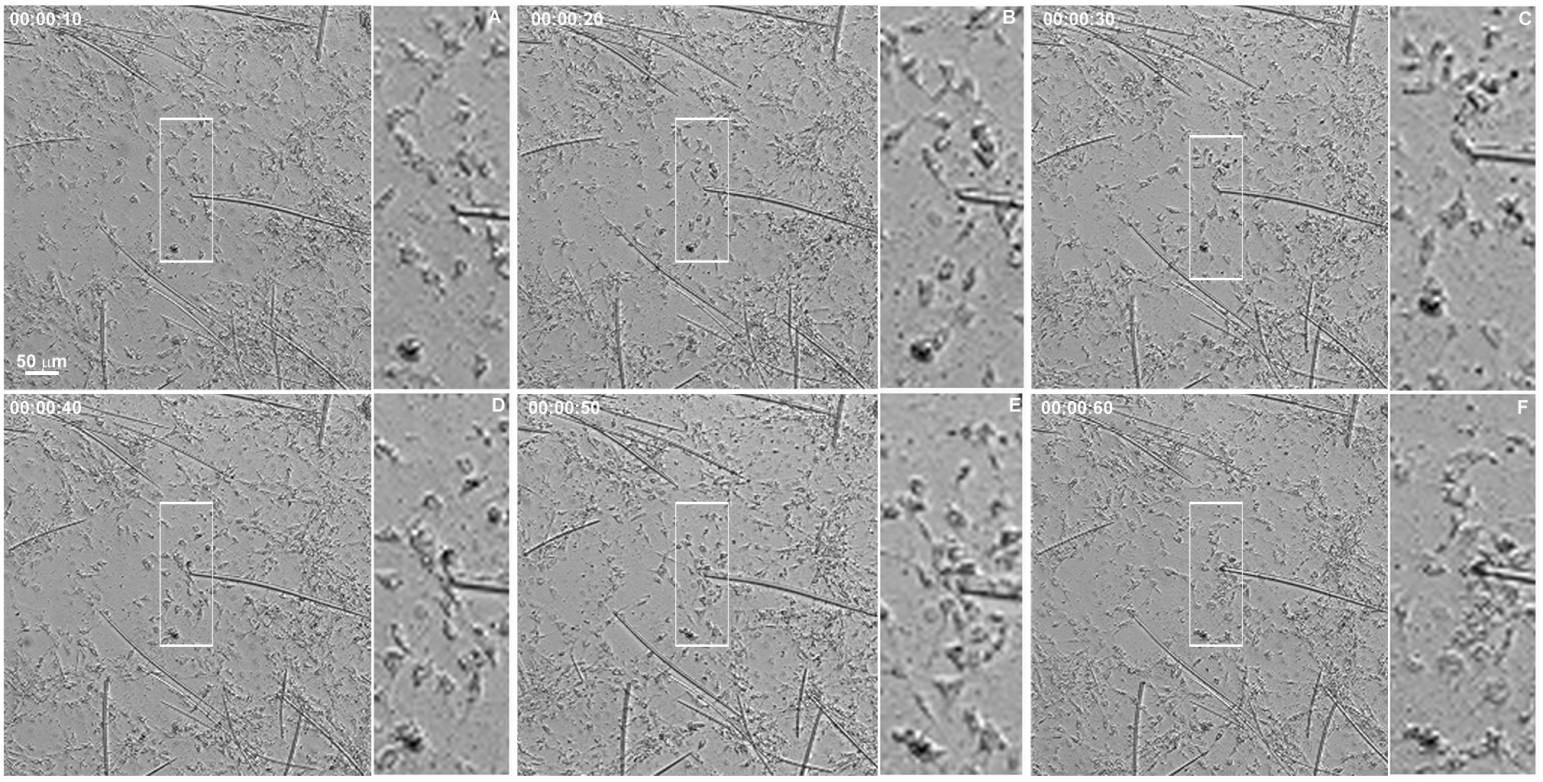
Individual mesenchyme-like cell migration in low cell density. Serial images from time-lapse video microscopy of the initial regeneration stage showing low density of cells with mesenchymal phenotype individually migrating for to form clusters. The white rectangle indicates a representative area in each image frame. The tip of the spicule inside the white square is the center of the indicated cluster. The regeneration time is shown in each stage (see S2 Fig).

**Fig 7:**
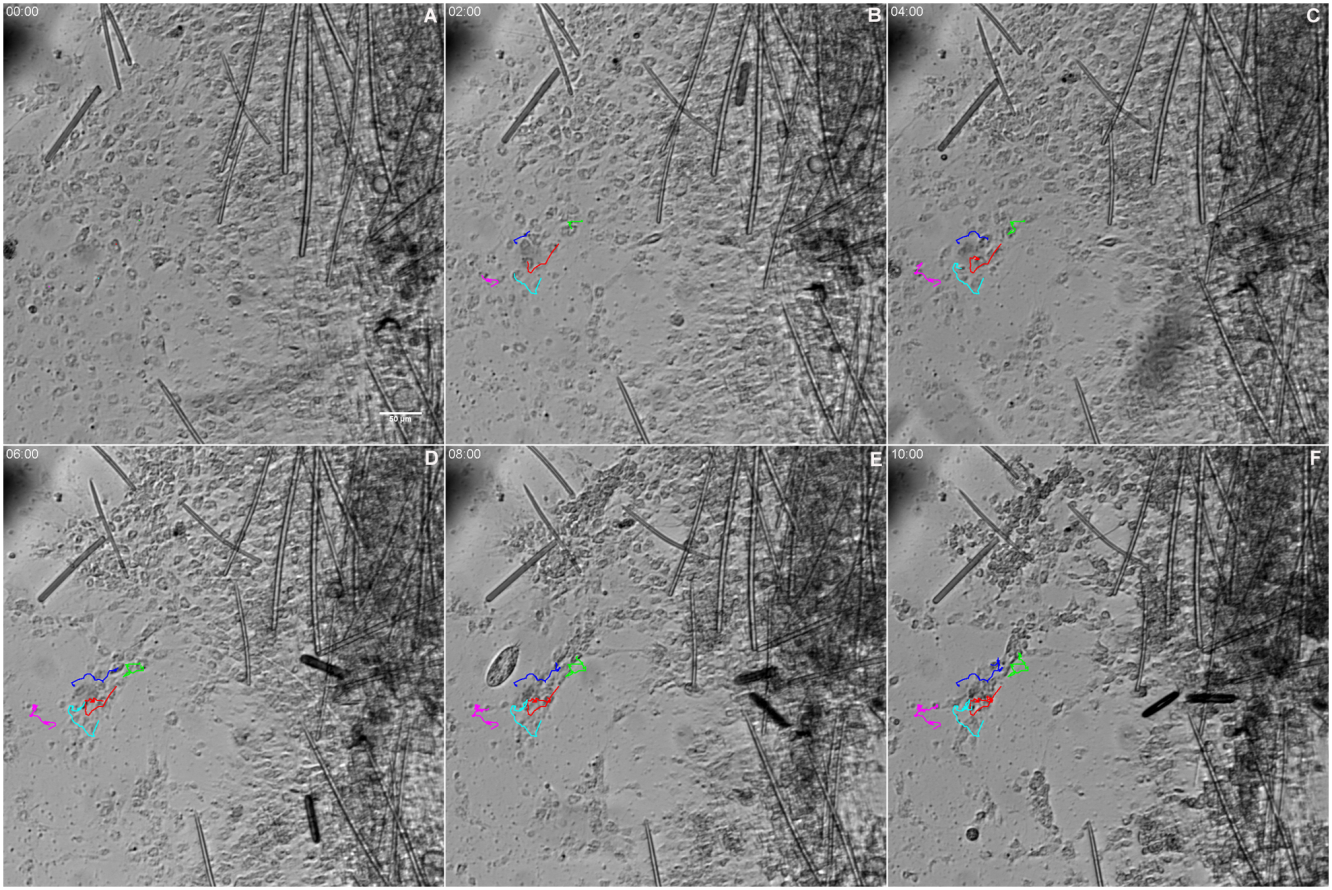
Initial flow of small groups of mesenchyme-like cells. Serial images from time-lapse video microscopy of the initial regeneration stage showing the formation of small groups of cells with mesenchymal phenotype streaming (arrows) toward the tissue with higher cell density. The regeneration time is shown in each stage (see S3 Fig).

**Fig 8:**
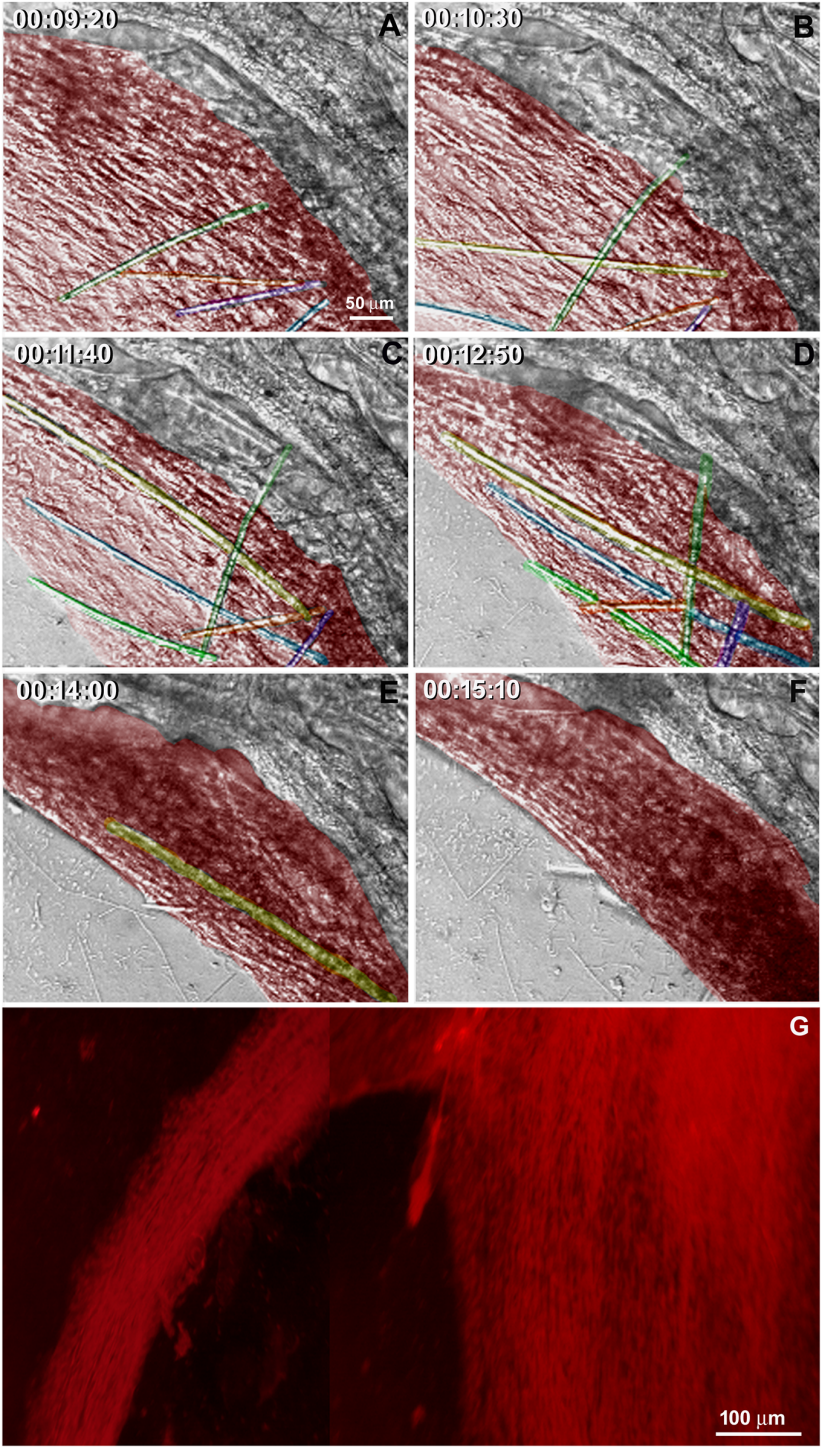
Tissue displacement in high density of mesenchyme-like cells. **A-H)** Serial images from time-lapse video microscopy of the initial regeneration stage showing high number of cells with spherical (nonaligned) morphology that are progressively pushed by fusiform mesenchymal cells forming a tissue stream (red). Spicules are identified by different colors to show their displacement and alignment inside the stream of cells with mesenchymal phenotype. The regeneration time is shown in each stage. **I)** Micrograph of a 20-h culture stained by Picrosirius to show the aligned organization of the collagen fibers inside the cell stream region (see S4 Fig).

The regenerative process begins as soon as the coverslips are taken from the sponge explants and placed in Petri dishes with seawater. We monitored the cell dynamics and formation of a new sponge through time-lapse video microscopy during 6.7 h. An entire sponge tissue contraction was observed under low magnification (Fig 5 and S1 Fig). To investigate more accurately the sponge tissue contraction, several images were recorded in fields having different proportions of cells with epithelial and mesenchymal phenotypes, and with different magnifications. Cells with mesenchymal phenotype were the first to migrate (exhibiting a high motility comparing with epithelial cells), initially in an apparently disorganized pattern and later tending to cellular aggregation. Three types of migration over the basoepithelial layer were observed, and cell density appeared to be determinant: I) at low density, when cells with mesenchymal phenotype were dispersed, initial stochastic migrations were followed by migration to the nearest cell cluster (Fig 6 and S2 Fig); II) small group of cells, including basopinacocytes, flowed toward the nearby more dense tissue (Fig 7 and S3 Fig); III) all sponge cell mass from a tissue-specific region underwent displacement *en masse* toward a specific direction, like a blastema movement, with filopodia guiding cells at the front, like cytonemes, and predominance of oriented fusiform cells, spicules and collagen fascicles (Fig 8 and S4 Fig). The data suggest that blastema formation in *H*. *heliophila* involves different centripetal movements depending on cell density, and is a consequence of a coordinated multicellular process, with morphological evidence of cell signaling.

**Fig 9:**
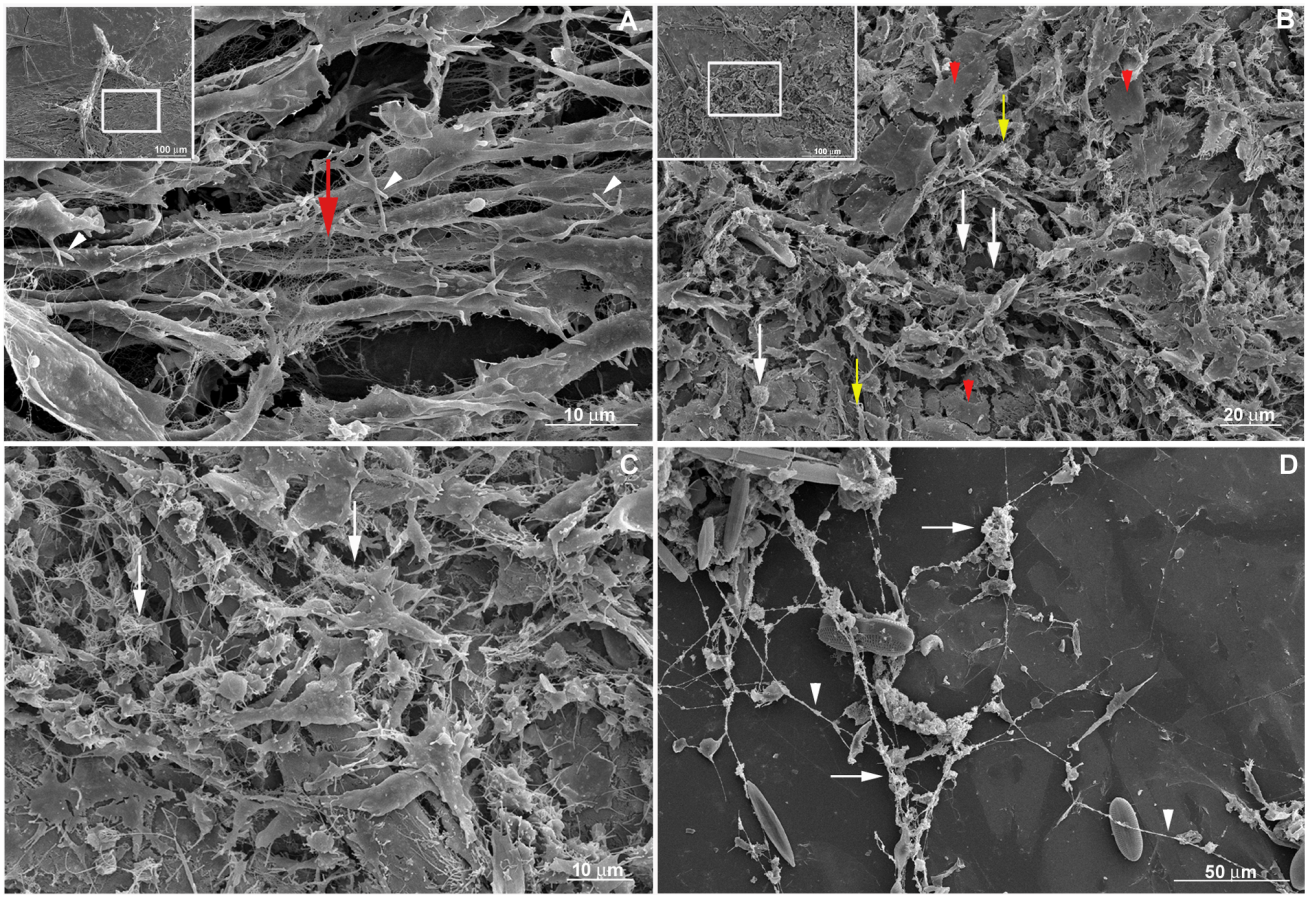
Tissue morphology at the initial stage of regeneration (T9hrs). **A)** Magnification of the area with cell stream marked at the inset image on the top left. Fusiform cells predominate in regions with cell stream. Filopodia are observed in all cells (white arrowheads). A representative area with collagen network of thin interlaced collagen fibrils organized into firm tracts is indicated by a red arrow. **B)** Magnification of the area marked at the inset image on the top left. Tissue with spherical cells predominating (white arrows) and some individualized epithelial cells (red arrowheads indicating a representative structure) and fusiform cells (yellow arrows for some of them). Filopodia are observed in almost all cells. **C)** 3D network of thin interlaced collagen fibrils organized into firm tracts (white arrows), that surround epithelial and mesenchyme-like cells. **D)** Long extracellular fibers (white arrowhead) with mesenchyme-like cell aggregation, debris (white arrows) and no epithelial cells.

Next, we investigated by SEM the cell migration and tissue organization after 9 h regeneration under culture conditions (Fig 9). Cell morphology ranged from spherical to fusiform, with some intermediate types. The proportion of these two cell types was unequal, forming distinct sponge tissue regions. Fusiform cells were more numerous within regions of tissue flow (Fig 9a), but could be observed moving independently in regions outside the flow (Fig 9b and 9c) as previously observed by time-lapse video microscopy for cell movement. These fusiform cells had filopodia, which varied in number and in length. Spherical cells predominated in certain regions of the main sponge tissue (Fig 9b and 9c). Variable numbers of filopodia were also observed in spherical cells.

The two types of extracellular fibers mentioned above might be playing a role during the aggregation process for blastema formation. When basal epithelium was no longer observed, long extracellular fibers remained with sponge cells aggregating alongside them (Fig 9d). The second type of fiber was observed in regions with higher cellular density forming a 3D network of thin interlaced collagen fibrils organized into firm tracts, to which spherical and fusiform cells were adhering (Fig 9a, 9b and 9c).

**Fig 10:**
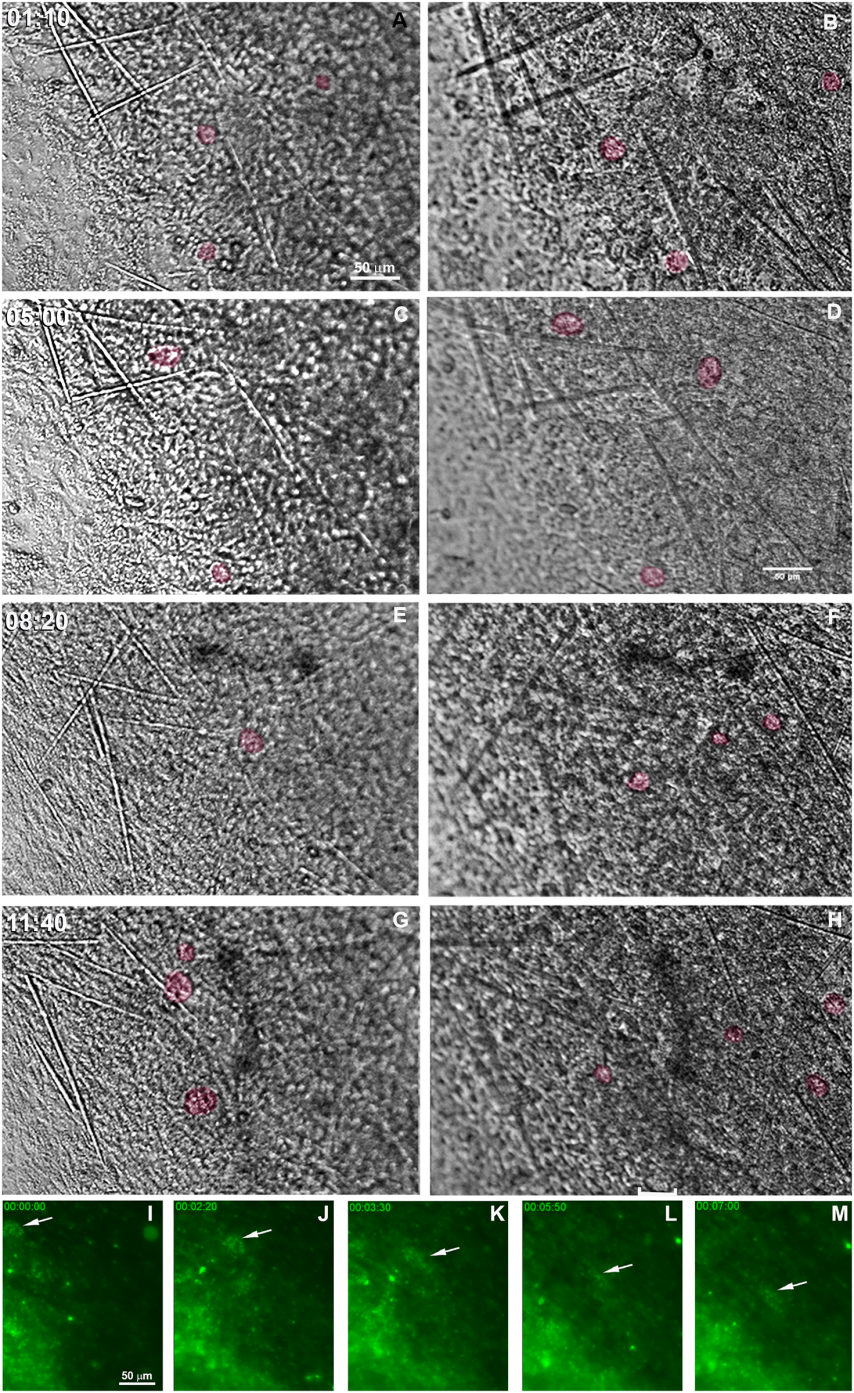
Persistence of some choanocyte chambers during the initial stage of regeneration. **A-H)** Serial images from time-lapse video microscopy of the initial regeneration stage taken from 2 focal plans. Each focal plane is shown in a column. Some representative choanocyte chambers are colored to show persistence after 11.7 h. The regeneration time is indicated for each stage. **I-M)** Serial images from time-lapse video microscopy of the initial regeneration stage of the endosomal tissue stained with vital staining Hoechst. The arrows indicate disorganization of choanocyte chambers after 3.5 h, and maintenance of the close contact among the choanocytes. The regeneration time is shown in each stage (see S5and S6 Figs).

Choanocytes, endopinacocytes and basopinacocytes appeared to differ from one another in their behavior during the initial stage of regeneration. Choanocyte chambers had their spherical structure preserved for different lengths of time, ranging from 1 to nearly 12 h. They were observed being pushed by the flow movement, sometimes with spinning movements (Fig 10 and S5 Fig). Hoechst nuclear vital staining was used to follow choanocyte chambers. These images showed the choanocyte chambers losing their spherical organization (Fig 10i–10m and S6 Fig). Despite the morphological changes, choanocytes remained in close contact with each other and did not disaggregate during the recording period.

**Fig 11:**
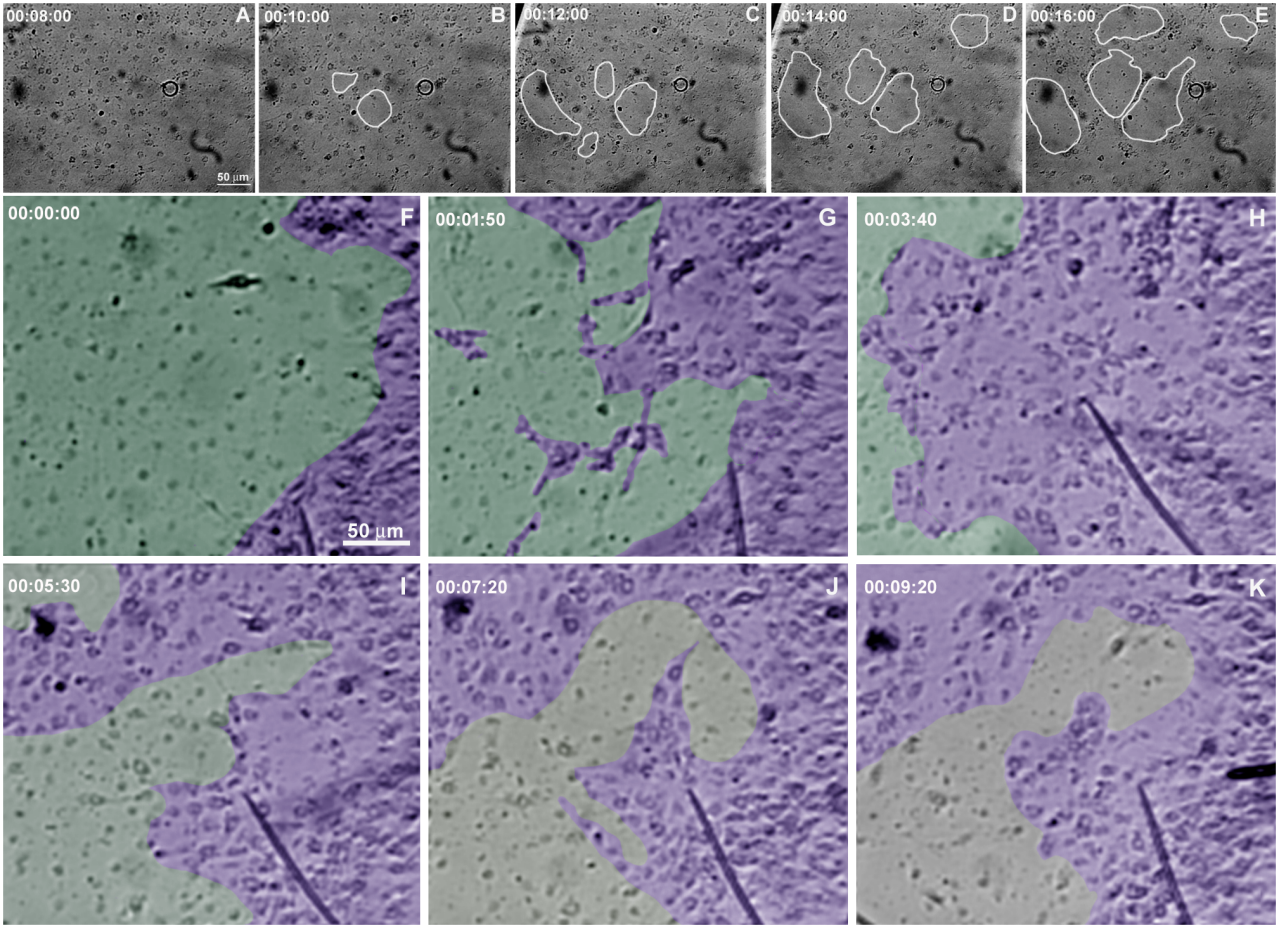
Basoepithelial to mesenchymal transition. **A-E)** Serial images (T0-T9 hrs) from time-lapse video microscopy of the basoepithelial to mesenchymal transition in a basopinacocyte-rich region. The homogeneous distribution of the basopinacocyte nuclei (white arrows in **A**) is visible at the initial stage. White circles illustrate the progressive increase of gaps in the basoepithelial layer. **F-K)** Serial images from time-lapse video microscopy of basoepithelial to mesenchymal transition in a basopinacocyte (green) and cells with mesenchymal phenotype (purple)-rich region. The homogeneous distribution of the basopinacocyte nuclei (white arrows in **F**) is visible at the initial stage (**F**) but is no longer visible at the final stage (**K**, gray area). Mesenchyme-like cell streams are formed at the final recording stage. The regeneration time is shown in each stage (see S7 and S8 Figs).

**Fig 12:**
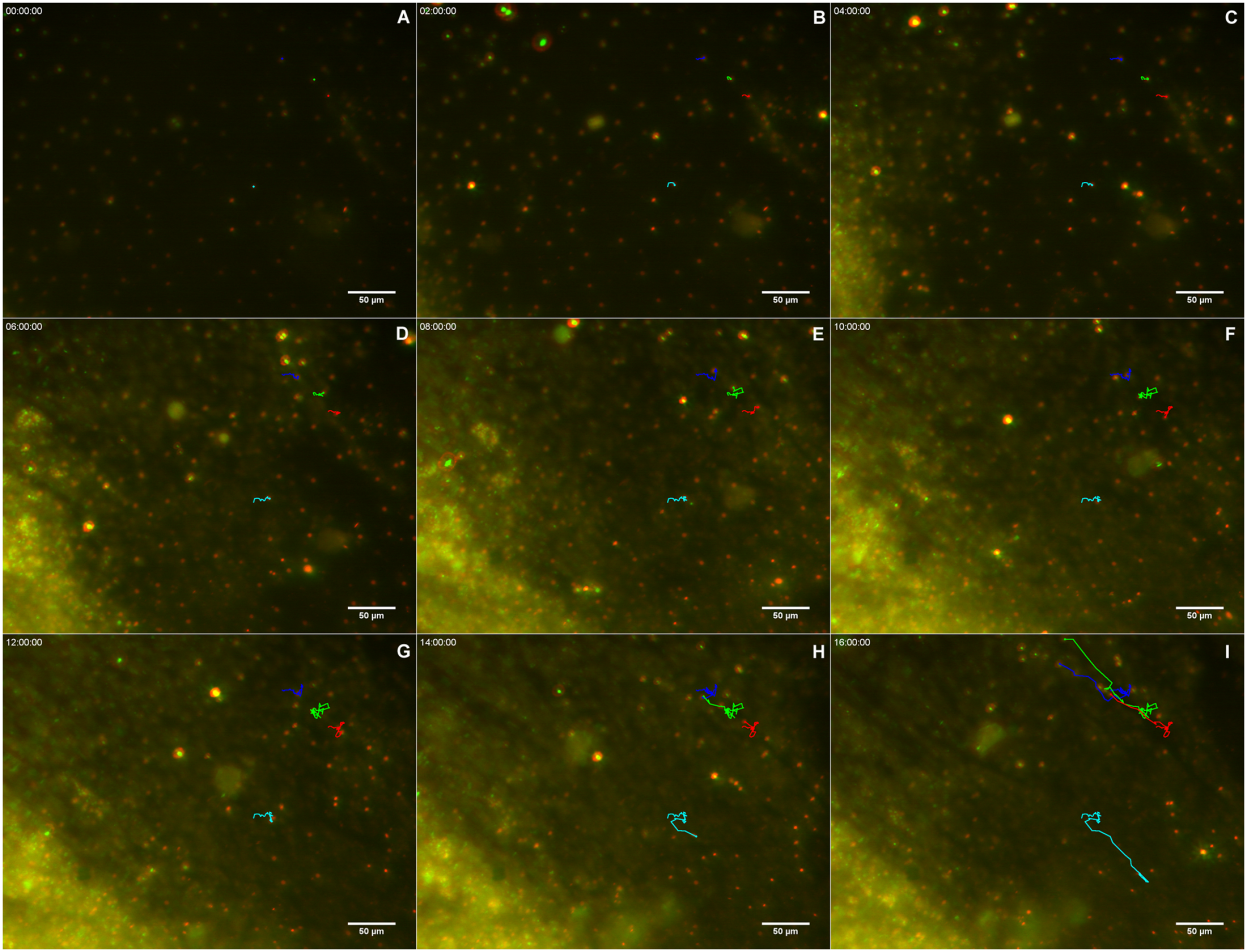
Basopinacocytes remain basal after 4 h mesenchymal transition. Serial images of Hoescht-stained nuclei in two different focuses, red for basoepithelial layer and green for superficial mesenchymal stream. Four basopinacocyte nuclei were manually tracked, and their route labeled (A-I) with the corresponding color to show cell displacement. Most cells with red nuclei converted to mesenchymal morphology (as it is supported by their displacement) without change of optical layer (red-to-green). The regeneration time is shown in each stage (see S9 Fig).

Analysis by time-lapse video microscopy recording in a field containing predominantly basopinacocytes suggested that cells were undergoing an epithelial/mesenchymal transition (EMT) and a clustering process (Fig 11a–11e and S7 Fig). To test whether EMT was modulated by the interaction of epithelial cells with mesenchyme-like cells, the regeneration process of other regions containing both cell types was recorded (Fig 11f–11k and S8 Fig). Basopinacocytes converted into mesenchymal phenotype in all the tested conditions, participating in the high-cell-density tissue flow and in the low-cell-density aggregation process. Most migrating cells converted from the basopinacoderm did not migrate toward the mesohyl, but retained their basal position during the tissue flow, as shown by distinctly different colors for the basal and mesenchyme-like cell layers (Fig 12 and S9 Fig).

**Fig 13:**
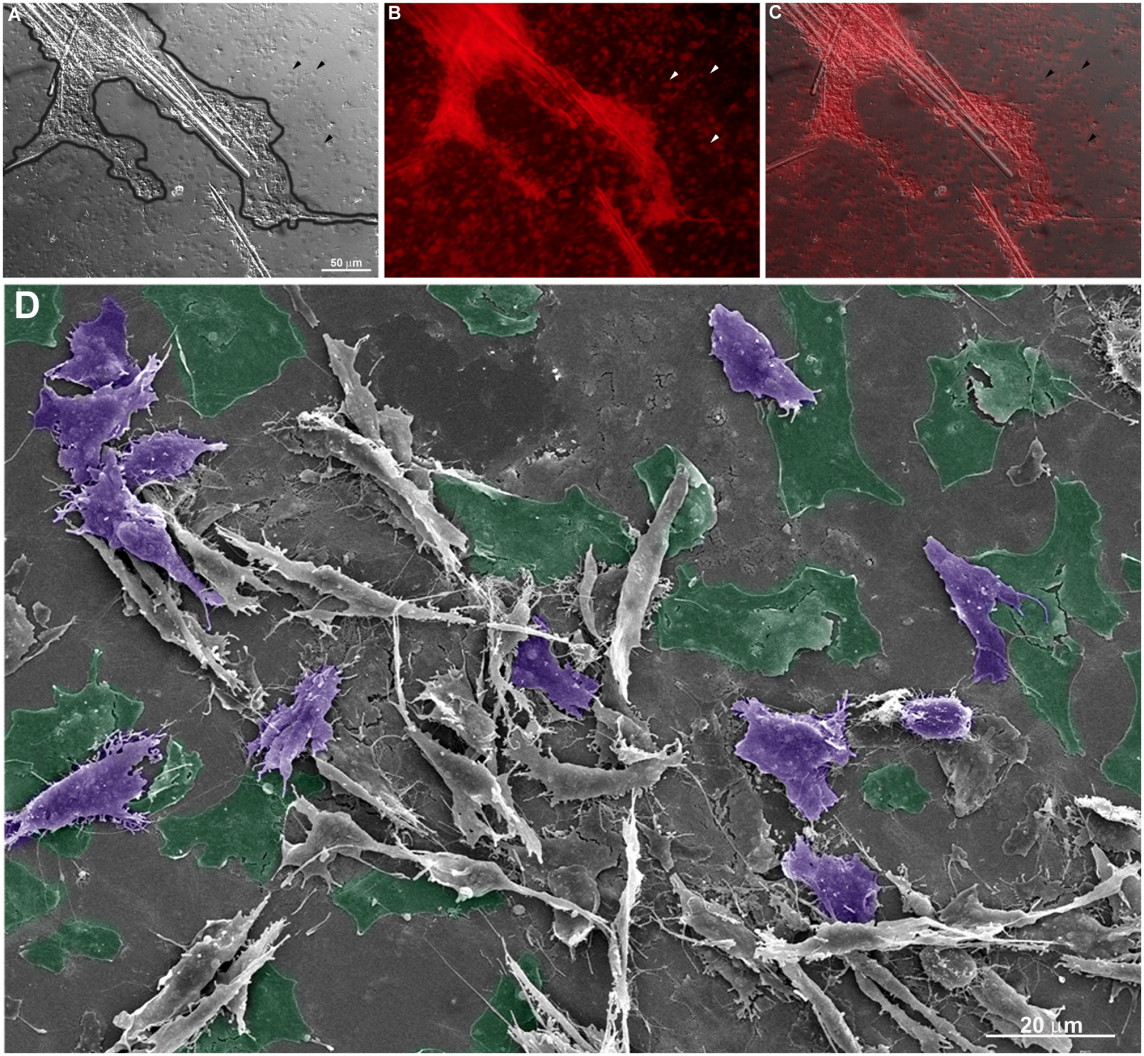
Evidence for basopinacocytes transition from a 9-h regeneration culture observed by SEM and Picrosirius staining. **A)** Differential contrast photomicrography of an endosomal region with cells with mesenchymal phenotype (outlined in black). Basopinacocytes already converted and migrated as mesenchymal cells after 9 h in culture (arrowheads point to the remaining collagen footprints). **B)** Picrosirius staining of the same field shown in “**A**”. **C)** Merged image from “**A**” and “**B**” showing the collagen nature of the basopinacocyte footprint (arrowheads). **D)** Putative field with epithelial-mesenchymal transition. Preserved basopinacocyte is shown in green and putative pinacocyte converting to mesenchymal morphology is colored in purple.

**Fig 14:**
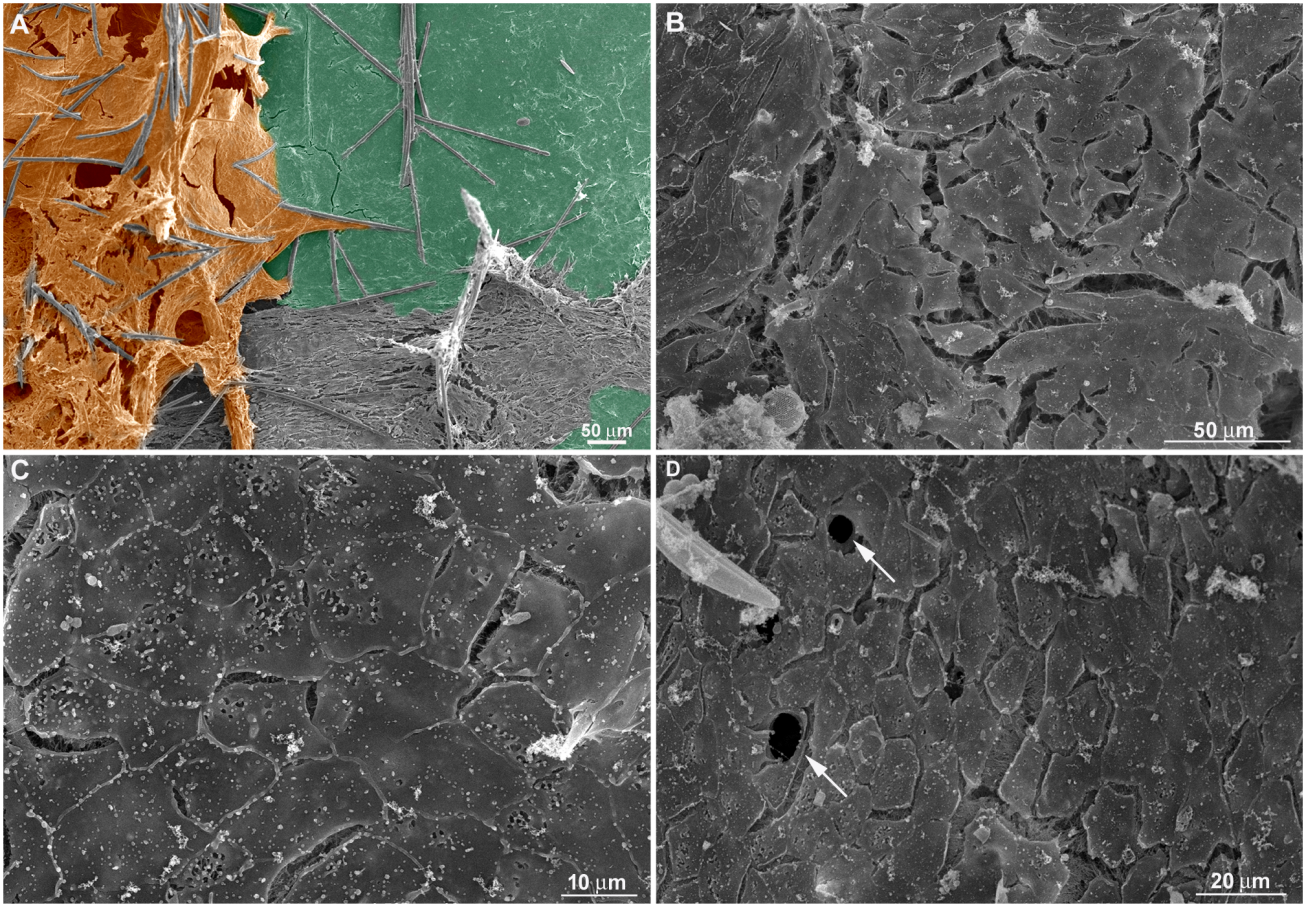
Superficial epithelialization process. **A)** A continuous epithelial layer of adjacent basopinacoderm (green) and superficial-covering pinacoderm (orange) was formed after 9 h of regeneration. Spicules are shown in blue. **B)** Nine-h regenerating culture showing incomplete superficial epithelialization. **C)** Three-day regenerating culture showing complete superficial epithelialization. **D)** Three-day regenerating culture showing complete superficial epithelialization and porocytes forming ostia (arrows). The observed gaps between the pinacocytes could be due to artifacts of fixation (**B-D**).

As observed by SEM and Picrosirius staining in some coverslip regions with a viable basopinacoderm, there was evidence of an epithelial/mesenchymal transition and basopinacocytes with migrating morphology (Fig 13a–13d). All stages for basopinacocyte morphological transition, from flattened adhered on the substrate (coverslips) to stream-forming mesenchymal morphology, were noticed after 9 h of regeneration. Basopinacocytes were more distant from each other, compared with T0, thus not forming a basal continuous layer and lacking cell-cell contacts. These observations provided additional evidence for EMT. The preserved border suggested minimal artifact from a possible fixation failure. None of the filopodia-rich cells displayed flattened epithelial morphology, indicating a navigating system after EMT. In some regions only the basopinacocyte footprints lasted (Fig 13a–13c). A continuous epithelial layer of adjacent basopinacoderm and their overlying pinacoderm layers were observed by SEM (Fig 14a). Morphological evidence for an epithelialization process were a distinctive characteristic of this developmental step (Fig 14b). Epithelialization of the wound surface was expected as part of the regenerative developmental program [17]. Complete superficial epithelialization with close cellular junctions and numerous porocytes, each one forming an ostium, was observed in three-day cultures (Fig 14c and 14d). The small gaps on the pinacoderm suggested small artifacts from a possible fixation problem.

### Dissociated cells inhibited tissue migration

**Fig 15:**
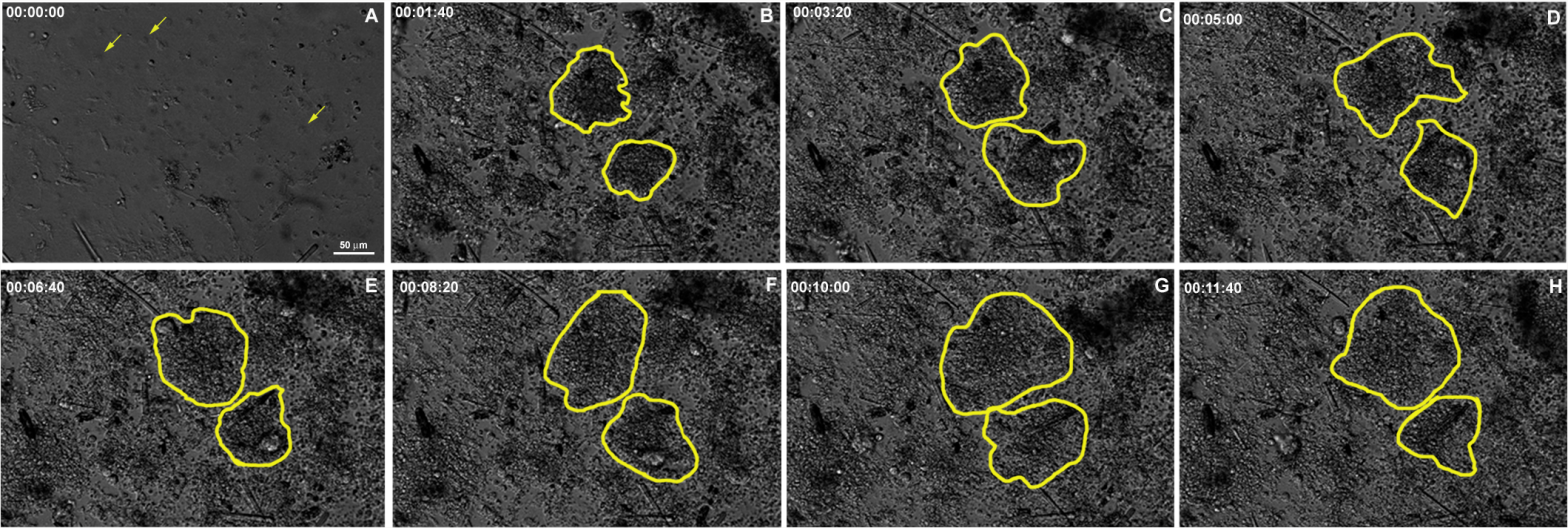
Time-lapse video microscopy with addition of dissociated cells. Dissociated cells were added over a basopinacoderm layer (**A**). Yellow arrows indicate representative basopinacocyte nuclei. **B-H**) Two initial cell clusters from incomplete dissociation are identified (yellow outlines). Cells of these two clusters dispersed progressively, as evidenced by the enlargement of marked areas and lightening color, from black to gray. The regeneration time is shown in each stage (see S10 Fig).

**Fig 16:**
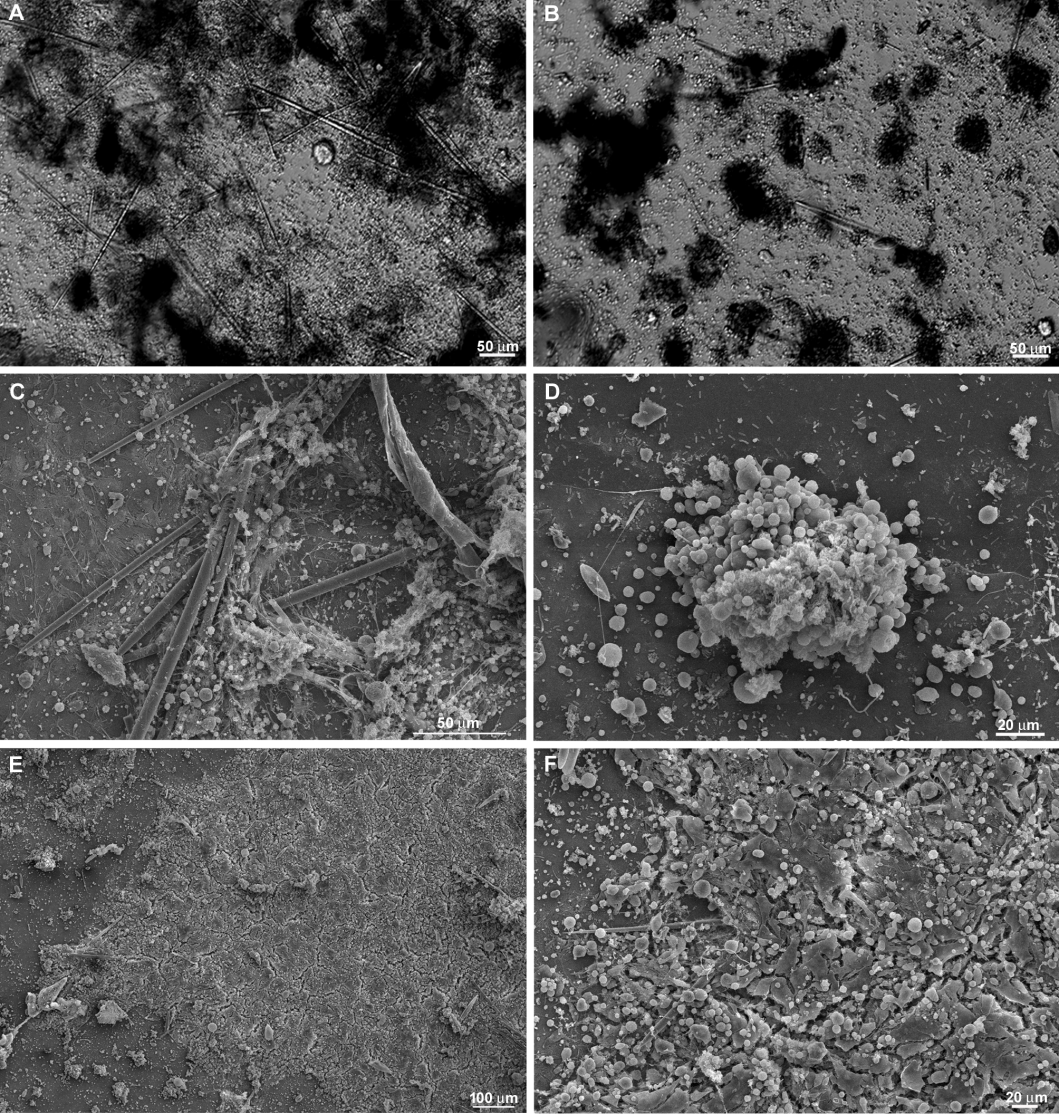
Dissociated cells integrate into the regenerating tissue and fewer clusters are formed. **A)** Differential contrast micrograph of a 20-h culture with addition of dissociated cells over a regenerating tissue. The cell-dense regions (dark gray and black) are not spherical. These high-cell-density areas were formed from previous regenerating tissue, indicating less cell aggregation. **B)** Cell clusters with spherical morphology (black) were formed in regions without previous regenerating tissue. **C)** SEM of a 20-h culture with addition of dissociated cells over a regenerating tissue. No spherical cell clusters were observed in this representative figure. **D)** SEM of a cell cluster formed in a region without regenerating tissue. **E)** Representative micrograph of 20-h regeneration after addition of dissociated cells showing advanced epithelialization and absence of fusiform cells. **F)** Higher magnification of a field shown in **E**.

Our results suggested interaction between cells with mesenchymal phenotype and basopinacocytes. Contact may promote signaling processes influencing EMT and the type of sponge cell movement, from individual cells to tissue migration. To test the involvement of cell-cell interaction in the regenerative process, freshly dissociated sponge cells were added to a sponge tissue in an initial phase of regeneration (T0). After this addition of new dissociated cells, time-lapse video microscopy revealed a distinct regenerative process. Newly dissociated spherical cells participated actively in the regenerating tissue, becoming integrated with the basoepithelium (Fig 15 and S10 Fig). Cell migration was observed in most experiments, but with a less frequent formation of cell clusters and without clear preferential direction. Control areas without sponge tissues attached to the coverslip had more cell clusters, as shown by Differential Interference Contrast microscopy (DIC) and SEM (Fig 16a–16d). The data support cell-cell interaction for regeneration. Moreover, SEM images suggested changes in regenerating sponge tissue 9 h after the addition of new dissociated cells. Epithelial cells were observed covering the tissue, without uncovered regions, but still gaps between pinacocytes (Fig 16e and 16f). These features suggest that superficial epithelialization was more advanced with than without addition of dissociated cells, an indication that these cells actively promote the regenerative developmental program. The observed gaps between the pinacocytes could be explained by uncompleted epithelialization, as expected for 9hs of regeneration time, or artifacts caused by the fixation procedure.

## Discussion

*Hymeniacidon heliophila* has morphological and ecological features suggesting high capability for regeneration [27] and resistance for the *in vitro* experimental procedures. The present article reports the regeneration process of an internal portion of *Hymeniacidon heliophila* organotypic culture. The tissue culture described here was used for time-lapse video microscopy using a confocal optical microscope, cell tracing, histology, SEM and cell transplantation. The observation of the sponge cells during the first 20 h of *in vitro* culture revealed different types of cell morphological transition, movements and cell-cell interactions, as part of the initial steps for mature sponge tissue recovery. These steps were similar to those described by Borisenko and colleagues [17], but migration of internal cells (epithelial and mesenchymal), epithelialization at the external surface (ectosome formation) and blastema formation were simultaneous in our regeneration model. The massive cell movements that occur during tissue displacement contrast with the localized cell migration described by Borisenko and colleagues [17]. The extent of sponge ablation may explain the differences seen here, as shown for the coral *Favia favus* [32] and discussed by Alexander and colleagues [33]. In our model, only a microscopic region of the internal portion of the sponge was attached to each coverslip; this was sufficient to initiate regeneration for complete sponge formation on the coverslip. Significant remodeling was necessary and cell aggregation with tissue contraction and blastema formation, as well as epithelialization initiation, occurred nearly simultaneously.

As a reference control, mature sponge histomorphology is shown here by HE and Picrosirius staining of histological sections. As usually observed in this type of sponge [16], the choanosome skeleton was truly confused and disorganized. Fusiform and spherical cells filled the mesenchymal space (mesohyl) between epithelial layers. Collagen forming fascicles and interlaced collagen fibrils organized into firm tracts with diffuse organization inside the mesohyl was confirmed by Picrosirius staining and SEM.

Regeneration began when the explant was opened and the coverslip was removed with its attached sponge internal tissue. The attached sponge tissue had various cell layers spread across the coverslip, which allowed for observation of distinct cellular processes according to cell density and cell type. As previously observed by Simpson [29], cell aggregation resulting in sponge body contraction with loss of feeding structures (reduction body) is a common stress response in sponges and was observed in this study. Moreover, time-lapse video microscopy and SEM allowed us to distinguish different cellular processes according to cell density, such as individual cells with mesenchymal phenotype emitting filopodia and pseudopods; small flow of cells; and massive cell flow with filopodia-rich cells at the front, guiding displacement of the entire tissue. Fusiform cells predominated in cell flows and spherical cells were part of the immobile sponge tissue, revealing distinct morphology according to the ongoing cell process. Isolated flattened cells observed by SEM at the mesohyl had a more irregular surface and more membrane projections, as compared to flattened cells forming a continuous epithelium. These features suggest a quorum sense and a navigating system for mesenchymal and flattened cell migration, probably performing blastema formation and superficial epithelialization to construct a new ectosome over the preexistent mesohyl/choanosome, thus forming a new sponge, as previously observed by Simpson [29].

Cells with mesenchymal phenotype that were rich in filopodia predominated over the basopinacoderm regions or guiding mesenchymal cell flow, as shown by time-lapse video microscopy and SEM. Cells with mesenchymal phenotype did not make intimate contact with the coverslip; instead they were confined to a layer that covered the underlying basopinacoderm or was attached to collagen fibers. The abundance of filopodia and restricted cell contact constitute evidence for apotactia, with specific cell adhesion and cell-transducing signals. This identifies basopinacoderm and collagen as specific contact and instructive inductors for internal organization.

Two types of extracellular matrix organization were observed by SEM. One type consisted of interlaced collagen fibrils organized into firm tracts covering the coverslip, spicules and between cells. This collagen matrix is probably the same as that reported in Pozzolini and colleagues [34] as a component of the cement responsible for basopinacocyte adhesion. The use of coverslips as substrate is probably inducing basopinacocyte adhesion, since the production of collagen cement is induced by silica [34]. The other type of matrix was formed by thicker and longer fibers forming a loose net that interconnected several cells. Long fibers nucleated clusters of moving cells along their length and may possibly provide molecular cues to keep cells together on the road (apotactia). Regions with characteristics of small cell flows (small numbers of aligned fusiform cells) and massive tissue flows (regions with many aligned fusiform cells) were also observed by SEM. The 3D tract of extracellular elements in these regions with characteristics of cell/tissue flow may be emitting signals that generate a coordinate movement of the entire sponge tissue.

The choanocyte chamber is considered an epithelial structure and was entirely preserved when the nearby mesenchymal cells were at high density and not performing tissue flow, as observed by time-lapse video microscopy. In contrast, chambers were engaged in a spinning movement while being pushed by the tissue flow, together with the alignment of some spicules. This suggests that the collagen fibers were remodeling and not holding the chambers together. Time-lapse video microscopy with vital cell dyes showed that some choanocyte chambers engaged in tissue flow lost their spherical shape. The data are not conclusive, but suggest an epithelial to mesenchymal transition (EMT), as reviewed by Borisenko and colleagues [17]. Moreover, vital staining revealed that these cells stayed clustered, suggesting the maintenance of choanocyte specific cell adhesion after choanocyte chambers have lost their spherical morphology. It is not clear whether the loss of spherical morphology affects cell proliferative capacity, as previously observed by Alexander and colleagues [33]. Further investigation extending the recording time for more than 20 h is necessary to follow choanocytes after chamber morphological changes and to address cell proliferation during regeneration.

Two-day-old cultures showed complete superficial epithelialization with specialized structures, such as ostia. Epithelialization was more advanced when dissociated cells were introduced over the regenerating tissue. All cells with mesenchymal and epithelial phenotype converted to spherical morphology after dissociation from the sponge tissue. Even so, improvement of the epithelialization was observed when spherical cells were in contact with regenerating sponge. It seems likely that some subpopulation of dissociated cells performs a spherical/epithelial transition for epithelialization, as reviewed by Borisenko and colleagues [17]. Morphological transition is a common process in sponge embryogenesis [19] during metamorphosis [18] and regeneration [17]. This high plasticity contrasts with bilaterian cells and is a feature also shared with colonial choanoflagellates (flagellate/amoeboid) [35]. The data suggest epithelial/mesenchymal plasticity as a primitive feature that was evolutionarily constrained by increased complexity, leading to cell type determination and germ layer formation.

As shown by time-lapse video microscopy with vital staining, basopinacocytes may also undergo EMT and cell migration, but they tend to retain their basal position during flow, suggesting a limited capacity for migration. Basopinacocyte transition to mesenchyme-like morphology was observed in either low numbers of mesenchymal cells or when basopinacocytes were found at the base of a large number of mesenchymal cells undergoing tissued flow. In contrast with these results, EMT was not observed when dissociated cells were plated over the regenerating tissue, nor was there massive tissue displacement and predominance of fusiform cells, but small cell flows without net displacement. This supports the idea that the EMT was the initial step for aggregation and regeneration. Further investigation is necessary to address the cell-cell communication process for blocking basoepithelial transition and centripetal movements.

The dissociated sponge cells formed clusters when they were not in contact with regenerating tissue, contrasting with their homogenous organization over regenerating tissue, as shown by time-lapse video microscopy, differential interference contrast light microscopy (DIC) and SEM. Moreover, mesenchymal spherical and flattened epithelial cells predominated in the regenerating tissue when cells were added, and the absence of fusiform cells provided morphological evidence for the absence of flow. These data suggest that interaction among dissociated cells and the regenerating tissue inhibits cell clustering in both populations.

The sponge regenerative process described here corroborates previous investigations [9,12,17,29]. During the extensive regenerative process we observed, all cell types, with epithelial and mesenchymal phenotypes, moved according to cell densities and formed a cell mass immersed in a 3D tract of extracellular matrix. Meanwhile, the regenerating tissue was progressively covered by pinacocytes. This sequence is similar to the blastema formation described for metazoan regeneration or primmorph generation after sponge cell dissociation, since no differentiated structures for feeding activity (choanocyte chambers and internal water channels) were observed, there was no evidence for spicule formation. This blastema could generate a new sponge, with choanosomes, when incubated in an aquarium cultivation system (data not shown), confirming that the *Hymeniacidon heliophila* blastema may be an intermediate step for complete regeneration. Formation of a blastema-like structure is a common process in sponge biology, seen for example in the initial step of embryonic development (blastulation) [35,36], metamorphosis [18], asexual reproduction [17,18,27] and regeneration. The lack of sponge cell determination, at least in *Amphimedon queenslandica* [19], and the plasticity for morphological transition could be the sponge strategy for extensive remodeling, including the distinguished capacity for regeneration. Since blastema refers to a proliferating pluripotent cell mass and it is a common process in sponge remodeling, further studies on sponge blastema development are needed for progress toward biomass generation in sponge farms.

## Material and methods

### Sponge organotypic culture

*Hymeniacidon heliopohila* is an encrusting demosponge, commonly found at the intertidal zone of Guanabara Bay, Rio de Janeiro, Brazil, where it grows to 0.5–10 cm in diameter and develops a thickness of 0.5–20 mm. Explants from *Hymeniacidon heliophila* are accessible throughout the year and possess the characteristics of a highly regenerative species [27]. All the sponge explants were collected from the field (Urca beach, coordinates -22.942759,-43.160115). As schematically represented in Fig 2, the first step was to collect an explant and perform a cut at the base (1–3 cm thick), transversal to the erect chimneys, fistules and uneven digitations that rise from a few millimeters to 4 cm high. After, two juxtaposed coverslips were introduced within the cut. The two halves of each explant were tied together with the two joined coverslips inside, using a kite line to keep the coverslips inside. This sandwich of sponge and coverslips was suspended in the bay for at least four days on a Styrofoam float close to the point of collection. Transport to the lab lasted 40 min at 20°C. The experiment began when the explants were opened at the lab and the coverslips were recovered for investigation. Sponge internal tissue, including basopinacoderm, spreads over the coverslips on just one side, the one facing the sponge tissue, but not the other side where the two coverslips were in contact. This attached tissue initiates regeneration and is used here as a model for sponge regeneration.

### Time-lapse video microscopy

A coverslip bottom of 1.5 x 1 cm was mounted in a Petri dish of 100 mm diameter to allow observation of living sponges using an inverted optical microscope for at least 16.5 h. The Petri dish was filled with fresh seawater (50 ml) and the coverslip with the attached sponge tissue was immersed in it. Images were acquired with a disk confocal Olympus DSU mounted on an Olympus IX80 inverted microscope using appropriate filters for fluorescence. Images were acquired with a Hamamatsu Orca 285 camera (Hamamatsu, Japan). Separate focal planes, with varied intervals in the Z-axis, were acquired in some images and time-lapse recordings. Images were processed with Image J software, based on the public domain NIH Image program (developed at the U.S. National Institutes of Health and available on the Internet at http://rsb.info.nih.gov/nih-image/).

The sponge tissue was always deposited on the upper surface of the coverslip to allow mechanical intervention and better water circulation. The temperature of the room and the natural seawater used were kept at 18–20°C during time-lapse video microscopy. No feeding process or any treatment for the natural sea water was provided. Images of different optical layers (5 μm) from the same field of the sponge culture were taken automatically every 10 min during 16.5 h.

Some cells were labeled with the nuclear dye Hoescht 33342 (Thermo Fisher Scientific, USA) for 15 min at room temperature in a solution with Hoescht at a final concentration of 5 μg/ml in 1 ml of seawater. Hoescht is a cell-permeant nuclear dye that emits blue fluorescence when bound to DNA. Image recording was initiated right after dye labeling. Suspensions with dissociated sponge cells were in some cases added to the *in situ* culture and an initial control image was taken before cell labelling. Isolated mesenchyme-like cells were counted in a Neubauer chamber and 2x10^5^ cells were added gently over the sponge tissue. Sponge cell dissociation was performed as initially described by Custodio and colleagues [20]. Differential Interference Contrast (DIC) microscopy was used in some experiments.

To better visualize different focal planes, layers were manually separated using Image J software and the basal optical layer was colored by replacing the gray scale with a red scale (applying a “red lookup table”) and the upper layer with a green scale.

### Scanning electron microscopy

For scanning electron microscopy (SEM), *in situ* sponge cultures were fixed with 2.5% glutaraldehyde, 4% formaldehyde in phosphate buffer and seawater for 1 h, post-fixed with 1% osmium tetroxide in seawater for 40 min, dehydrated in steps to 100% ethanol and critical-point dried (Baltec CPD 050). Coverslips were placed on carbon tape covering aluminum stubs, gold-sputtered for 120 s (Balzer FL-9496) and examined using a Jeol JSM-5310, operating at 10 kV.

### Histological analysis

Coverslips with sponge tissue incubated for 0 and 20 h were fixed using Bouin´s solution (6 h). The samples were stained with Picrosirius, as originally described by Dolber and Spach [37] and with the nuclear dye DAPI (0.1 ug/ml in 0.9% NaCl). Preparations were examined in a disk confocal Olympus DSU mounted on an Olympus IX80 inverted microscope (Olympus) using appropriate filters for fluorescence.

### Nuclear staining

*In situ* sponge cells were rinsed with PBS and fixed with 4% paraformaldehyde in PBS for 10 min at room temperature. Cells were then washed once with 0.9% NaCl and nuclei were labeled with DAPI (0.1 μg/ml in 0.9% NaCl) for 5 min). Cells were mounted in ProLong Gold antifade reagent (Molecular Probes) and examined with an Axiovert 100 microscope (Carl Zeiss, Germany). Images were acquired in grayscale with an Olympus DP71 digital camera (Olympus, Japan) and displayed as a cyan scale (“cyan look-up table”).

## Supporting information

S1 Fig Cell movement during regeneration. (AVI)

S2 Fig Individual mesenchyme-like cell migration in low cell density. (AVI)

S3 Fig Initial flow of small groups of mesenchyme-like cells. (AVI)

S4 Fig Tissue displacement in high density of mesenchyme-like cells. (AVI)

S5 Fig Persistence of some choanocyte chambers during the initial stage of regeneration. (AVI)

S6 Fig Persistence of some choanocyte chambers during the initial stage of regeneration. (AVI)

S7 Fig Basoepithelial to mesenchymal transition. (AVI)

S8 Fig Basoepithelial to mesenchymal transition. (AVI)

S9 Fig Basopinacocytes remain basal after 4 h mesenchymal transition. (AVI)

S10 Fig Time-lapse video microscopy with addition of dissociated cells. (AVI)
